# The economic burden incurred by families caring for a young child with developmental disability in Uganda

**DOI:** 10.1371/journal.pgph.0000953

**Published:** 2023-04-19

**Authors:** Kenneth R. Katumba, Cally J. Tann, Emily L. Webb, Patrick Tenywa, Margaret Nampijja, Janet Seeley, Giulia Greco

**Affiliations:** 1 Medical Research Council/Uganda Virus Research Institute and London School of Hygiene & Tropical Medicine (MRC/UVRI and LSHTM) Uganda Research Unit, Entebbe, Uganda; 2 Department of Global Health and Development, London School of Hygiene and Tropical Medicine, London, United Kingdom; 3 Department of Infectious Disease Epidemiology, London School of Hygiene and Tropical Medicine, London, United Kingdom; 4 Neonatal Medicine, University College London Hospitals NHS Trust, London, United Kingdom; BRAC James P Grant School of Public Health, BRAC University, BANGLADESH

## Abstract

Each year, nearly 30 million children globally are at risk of developmental difficulties and disability as a result of newborn health conditions, with the majority living in resource-constrained countries. This study estimates the annual cost to families related to caring for a young child with developmental disability in Uganda. Nested within a feasibility trial of early care and support for young children with developmental disabilities, this sub-study estimated the cost of illness, the cost of paternal abandonment of the caregiver and the affordability of care by household. Seventy-three caregivers took part in this sub-study. The average annual cost of illness to families was USD 949. The main cost drivers were the cost of seeking care and income lost due to loss of employment. Households caring for a child with a disability spent more than the national average household expenditure, and the annual cost of illness for all households was more than 100% of the national GDP per capita. In addition, 84% of caregivers faced economic consequences and resorted to wealth-reducing coping strategies. Families caring for a child with severe impairment incurred USD 358 more on average than those with mild or moderate impairment. Paternal abandonment was common (31%) with affected mothers losing an average of USD 430 in financial support. Caring for a young child with developmental disability was unaffordable to all the study households. Programmes of early care and support have the potential to reduce these financial impacts. National efforts to curb this catastrophic health expenditure are necessary.

## Introduction

Annually, an estimated 30 million neonates require specialized facility-based care, with the survivors at increased risk of life-long conditions including developmental disabilities [1,2]. Common neonatal conditions associated with perinatal brain injury, lead to an increased risk of developmental disabilities, including cerebral palsy (CP), global developmental disability, visual and hearing loss and seizure disorders [1,3]. Caring for a child with developmental disability may have major economic and social impacts on families in any country, but this is potentially higher in lower- and middle-income country settings (LMICs) where support services are often few, and stigma may be more overt [4–11].

### The economic burden incurred by families caring for a child with developmental disability

The scalability and sustainability of programmes that improve quality of life, life chances and other health outcomes of children with developmental disabilities and their families is dependent on their cost and cost-effectiveness. Uptake of such programmes by families depends on the level of costs incurred, especially in LMICs where resources are scarce and existing care structures for affected children and adults often less well established [12–14]. Cost of illness (CoI) studies, from the user perspective, have been used to estimate the economic burden of diseases and conditions to patients and their caregivers, considering both tangible and intangible aspects of the disease or condition [15,16]. Those from the societal perspective provide an estimate of the potential economic savings society would make by adopting prevention campaigns [9,16]. To date, CoI studies for children and adults with developmental disabilities are few. Some studies have estimated the cost of caring for a child with developmental disability, including CP, from the societal perspective in high-income countries (HICs); the Centers for Disease Control and Prevention (CDC) reported the CoI of CP in the United States as 503,000 USD in 1995 [17] and 921,000 USD in 2003 [18]. Kruse et al. reported annual CoI in Denmark as 968,000 USD for men and 900,000 USD for women in 2009 [19], while Wang et al. reported CoI from China as 67,044 USD in 2008 [9]. Only Wallace et al. have published the estimated annual CoI related to childhood developmental disability from the provider perspective in the United States as 1,039 USD in 1995 [20].

No studies to date have directly reported costs to households, nor compared the costs by socio-economic status. All the studies that modelled lifetime costs reported higher CoI for children with more severe CP, and reported indirect productivity losses as the largest cost drivers [9,17–20]. Wang et al. used average expenditure and utilization rates for different services by age groups to estimate the lifetime economic burden related to CP from the societal perspective, categorizing costs as direct healthcare costs, direct non-healthcare costs, developmental costs and indirect costs [9]. Although they did not report CoI figures, some other studies used relevant costing methodology: Guyard et al. and Park et al. both documented time spent giving care as a major driver of the economic burden incurred by caregivers as a result of caring while Tonmukayakul et al. reported a strong positive relationship between the severity of CP experienced and expenditure [21–23]. All the studies estimating the economic impact of NDI or CP, documented both the direct and indirect costs to families and to the society [9–11,14,17–27]. Although some studies have examined the CoI associated with developmental disabilities in children in HICs [28], research from LMICs is lacking, including from sub-Saharan Africa.

### Research aims and objectives

This study aimed to estimate the annual cost of caring for a young child with developmental disability for Ugandan households. Our objectives were to i) estimate the total annual cost, ii) describe the drivers of these costs, and iii) investigate uniformity of annual costs across groups according to income and geographical location.

## Methods

### Ethics statement

This study received ethical approval from the Uganda Virus Research Institute Research Ethics Committee (GC/127/17/04/5), and from the Uganda National Council for Science and Technology (CRC/MN/1 HS 2244). Written informed consent for entry into the feasibility trial was sought and obtained from the caregiver (in English or Luganda, the main local language) and it was inclusive of consent for the cost of illness sub-study. Oral and written explanations of the economic evaluation sub-study were provided, and those who wished to withdraw their consent were given the opportunity to do so. Transport reimbursement was provided to respondents.

### Study setting

This study was conducted within the Baby Ubuntu feasibility trial, examining feasibility, acceptability and early evidence of impact of a programme of early care and support for young children (<2 years) with developmental disabilities and their caregivers [29]. The feasibility trial was a two-centre, single blind, randomized controlled trial with two parallel groups to evaluate this participatory, group, early care and support programme compared to standard care. The trial methodology has been previously described [30] and the findings reported [29]. Trial participants were young children (6–11 months of age) with neurodevelopmental impairment (NDI) consistent with a diagnosis of developmental disability and their caregivers, from two sites in Uganda: Mulago Hospital, the National Referral Hospital in Kampala; and Kiwoko Hospital in Nakaseke District, central Uganda [30]. Recruited children were 6–11 months of age and were followed to 18–23 months. Recruited participants underwent standardized scored neurodevelopmental assessments using the Griffiths Mental Development Scales-II (GMDS) to assign a developmental quotient (DQ) and the Hammersmith Infant Neurological Examination (HINE). NDI at 6–11 months was defined as a DQ<70 and, or HINE <60 [30]. Severity of NDI was categorized as mild (DQ 70-<85), moderate (DQ 55-<70) or severe (DQ <55).

### Estimating the cost to households of caring for a young child with developmental disability

We adopted a prevalence-based approach as stipulated by Jo et al. (2014) to estimate the economic cost to households, and according to the methods described by Wang et al. (2008) who included direct health care costs, direct non-healthcare costs, and indirect healthcare costs in their estimation of the CoI [9,31]. Over a three-month period, data from each household were collected and the results extrapolated to twelve months estimates. During feasibility trial recruitment (N = 126), we purposively sampled 94 participants for this CoI sub-study in order of attendance: 47 from the intervention and 47 from the control arm.

In line with the approach taken by others [9,19,31], we identified, collected, and estimated different categories of direct and indirect costs. As described in Table 1, these included: direct costs of seeking medical care; direct costs in the form of reductions in household wealth in order to accommodate the costs of caring, and any special expenses such as home modifications incurred to improve the wellbeing of the child affected by developmental disability; and indirect costs such as loss of employment and loss of income due to caring responsibilities [9,19,32]. Data were collected using 30–40 minute semi-structured questionnaires (S1 Text) administered by a trained research assistant. We collected information on the main caregiver’s background, employment, economic activities, number of household dependents and caregivers to the child. We considered only visits to the health facilities that were strictly related to the care of the child. Costs related to attending the trial intervention activities (Baby Ubuntu programme) were excluded except for those that coincided with healthcare appointments and hospitalization.

**Table 1:** Items considered to contribute to cost of illness for children with developmental disabilities.

Item	Costs included	
***Direct costs***		
Cost of seeking care	Inpatient admissions and outpatient visits	Transport to the facility (return) Admission fees Food and refreshments Drugs • prescribed and purchased within the facility • purchased outside the facility Laboratory tests • performed within the facility, • performed outside the facility Consultation and other fees.
Reduction in wealth	Direct reduction in physical wealth/assets as coping mechanisms due to the child’s illness calculated as the sum of the following: • Value of goods the household had to sell to seek and pay for care for the child • Total amount of money paid back after borrowing for the child’s health care • The reduction in the household’s food consumption.	
Special home modifications	Sum of expenses incurred due to special home modifications to accommodate the child’s condition.	
***Indirect costs***		
Income lost due to loss of employment	Loss of employment of the main caregiver and calculated as the product sum of the average monthly income and the number of months of work lost since the child was diagnosed. These were considered only where a caregiver reported to have lost work or laid off to care of a child.	
Income lost in time caring for child (income lost due to caring for child at a health facility and at home)	Caregivers’ income loss •Defined as income that the main caregiver lost due to absence from work and/or taking days off work to take care of the child. •calculated as the product sum of the average daily income and the number of days of absence and/or taken off work to care for a child.	
	Household members’ income loss. •Defined as the income lost by all household members that took time off their work to take care of the child. •Calculated as the product sum of the average daily incomes and the number of days of absence and/or taken off work to take care of the child.	

We calculated the cost of illness–the economic costs of caring for a young child with developmental disability—using the formula below.

#### Cost of Illness of caring for a child with developmental disability = Cost of seeking care + Income lost due to loss of employment + Income lost in time caring for child + Reduction in Wealth + Special home modifications

The estimation of each of the individual items in the formula is detailed in Table 1.

### Cost of abandonment

Paternal abandonment of the main female caregiver was recorded during the feasibility trial. In response, we estimated the cost of abandonment, defined as the direct net reduction in the main caregiver’s income resulting from the main caregiver (usually the child’s mother) being abandoned by their partner (usually the child’s father). Caregivers reported losses in their periodical financial support (whether for child support or the mother’s upkeep) since their child’s diagnosis, which was then utilized to calculate the cost of abandonment as the value of this loss over a one-year period.

### Socio-economic status and affordability of care

Household socio-economic status was estimated using average annual household expenditure on items other than rent. Rent/housing was excluded since homeowners do not pay rent meaning inclusion would negatively skew estimates of annual expenditure. We adopted the methodology used in previous studies on affordability and catastrophic expenditure for persons with disabilities, where expenditure on health is compared to household expenditure and determined to be catastrophic or unaffordable if it is more than 10–49% of household expenditure [33–36].

### Data collection and management

Data were entered into a MS Excel database by a trained research assistant with supervision from a health economist. Data validation using pre-programmed checks in the Excel sheets was carried out immediately, supplemented by weekly validation checks. The database was cleaned and exported to Stata SE 15, for analysis.

Cost data were collected in Uganda shillings and converted to United Stated Dollars (USD) using the average Bank of Uganda exchange rate for May 1^st^ 2018 –April 30^th^ 2019, the time of data collection [37]. We calculated descriptive statistics for costs both with missing values considered (overall) and restricted only to those families where the cost was reported, therefore not considering the families which had a missing value for that cost. The Kruskal-Wallis equality-of-populations rank test tested for the equality of means. The null hypothesis of equal means between groups was rejected if the p-value was <0.05.

We considered missing values to be missing completely at random as missingness did not correlate with other variable values in the dataset. Missing values for continuous variables (i.e., costs) were not imputed, but modes for missing qualitative variables, such as the ‘means of transport’, were.

## Results

### Study participants

The Baby Ubuntu feasibility trial recruited 126 children, with 82 from Mulago Hospital (urban site), and 44 from Kiwoko Hospital (rural site) [29]. Of these 63 were randomized to receive the intervention and 63 to standard of care.

Of the 94 participants selected for the CoI sub-study; 4 children had died; 12 had unreachable phone numbers; and 5 did not attend the planned interview meetings. We therefore interviewed 73 caregivers (72 females, 1 male) of 73 children (37 females, 36 males); 42 received the intervention and 31 standard care. Interviews were carried out between July 2017 and April 2019.

Data for three caregivers were excluded during analysis because of the extremely high costs of illness reported ($5,368, $9,126 and $12,979), much higher than their annual spending, and the national reported average expenditure ($1,044), indicating reporting error. Our data analysis therefore included data from 70 respondents. The baseline clinical characteristics of the children included in our analysis are described in Table 2 below.

**Table 2:** Baseline clinical characteristics of the included caregivers and children (N = 70).

*Baseline characteristics*	*n*
***Caregiver characteristics***	
Sex	69 female, 1 male
Living context	42 urban, 28 rural
***Child characteristics***	
Child sex	36 female, 34 male
Age in months, mean (SD)	15.6 (4.9)
Developmental impairment, by severity	
Overall global DQ, median (%) [IQR]	31.6 (100) [24.7–34.6]
Mild	72.1 (4.3) [71.9–75.5]
Moderate	65.7 (5.3) [57.0–67.6]
Severe	28.9 (90) [20.2–33.5]
Neurological impairment, by severity	
Overall HINE score, median (%) [IQR]	36.0 (100) [28.5–42.0]
Mild	64.0 (5.7) [60.0–69.0]
Moderate	46.5 (35.7) [44.1–48.0]
Severe	25.5 (58.6) [21.5–28.5]
Feasibility trial arm allocation	40 intervention, 30 standard care

SD = Standard deviation, DQ = Developmental Quotient, IQR-Interquartile range, HINE-Hammersmith Infant Neurological Score.

### Total annual cost of caring for a child with developmental disability and relative affordability

Table 3 reports on the total cost of caring for a child with developmental disability and its components. Lowest household cost was USD 10, whilst the highest cost was USD 3,921 and both were from the urban site (Mulago National Referral Hospital). The household that spent USD 10 reported reducing their food consumption since their infant (1 year 4 months) had been diagnosed with developmental disability but had no other expenditure related to their child’s condition. The household that recorded the highest cost had relatively higher costs across all the cost categories than other households, except ‘Income lost in time caring for child’. Furthermore, 70% of the caregivers in the sample faced impoverishing economic consequences as shown by their selling of personal property (43%), their reduction of essential food consumption (41%), and borrowing of money (41%) to provide care for their children.

**Table 3:** The total annual cost of caring for a young child with developmental disability in Uganda (USD ($)) (N = 70).

		Average annual cost, among those reporting this cost		Average annual cost, among all participants	
Variable	Number (%) reporting this cost	Median (range) $	Mean (SD) $	Median (range) $	Mean (SD) $
***Cost of seeking care***	69 (99)	303 (11, 2628)	528 (588)	302 (0, 2628)	521 (587)
***Income lost due to loss of employment***	20 (29)	487 (23, 3245)	666 (723)	0 (0, 3245)	190 (485)
***Income lost in time caring for child***	6 (9)	233 (135, 292)	222 (72)	0 (0, 292)	19 (66)
*Income main caregiver lost due to days off work*	3 (4)	195 (146, 292)	211 (74)	0 (0, 292)	9 (45)
*Cost of someone else hired to cover mother’s work*	1 (1)	292 (292, 292)	292 (0)	0 (0, 292)	4 (35)
*Income lost by household members covering mother*	2 (3)	203 (135, 270)	203 (96)	0 (0, 270)	6 (36)
***Reduction in wealth***	49 (70)	81 (10, 1766)	253 (392)	32(0, 1766)	177 (346)
*Value of foregone food*	29 (41)	16 (1, 487)	66 (121)	0 (0, 487)	27 (83)
*Value of assets sold*	30 (43)	76 (11, 1717)	267 (448)	0 (0, 1717)	114 (319)
*Amount of money borrowed and returned*	29 (41)	41 (5, 811)	85 (153)	0 (0, 811)	35 (106)
***Special home modifications***	40 (57)	97 (3, 837)	154 (200)	10 (0, 837)	88 (169)
***Total cost of illness***	70 (100)	597 (10, 3921)	949 (898)	597 (10, 3921)	949 (898)

### Income and wealth lost due to caring

Twenty caregivers reported to have lost a mean of USD 666 (range: 23–3245) due to loss of income. Six participants reported losing a mean household income of USD 222 (range:135–292) due to time taken off work to take care of their child.

### Cost of illness by severity of disability

The role of severity of developmental disability in cost of illness was also examined. Children with severe neurological impairment on the HINE score (<40) had on average USD 358 higher costs than less impaired children (p = 0.0503). No significant difference was seen between severity groups based on DQ although power for this comparison was limited as the number of participants with mild-moderate impairment based on DQ was small (n = 7).

### Cost of caring for a child with developmental disability by urban and rural sites

Table 4 reports the cost of care by urban and rural sites. The proportions reporting each type of cost were similar between urban and rural settings. The reported mean costs are the means among only those households that incurred those costs. Households in urban areas spent significantly more on average in a year than those households in rural areas (USD 1,132 vs USD 674 respectively). The biggest driver of this difference was wealth reduction: selling household assets, borrowing money, and reducing food consumption. Urban households on average lost wealth worth USD 359 while rural households lost wealth worth USD 202. When the analysis was carried out with the missing values considered, and with the mean costs compared between rural and urban areas, the difference in wealth reduction became insignificant.

**Table 4:** Rural vs urban annual cost (USD $) of caring for a young child with developmental disability.

	Rural site (Nakaseke), N = 28			Urban site (Kampala), N = 42				
***Variable name***	**Number (%) reporting this cost**	**Mean annual cost of illness** **(range)**	**SD**	**Number (%) reporting this cost**	**Mean annual cost of illness(range)**	**SD**	**Kruskal-Wallis H**	**P-value**
Cost of seeking care	28 (100)	486 (28, 2628)	592	41 (97.6)	557 (11, 2479)	623	0.03	0.871
Income lost due to loss of employment	1 (3.6)	195 (195, 195)	0	5 (11.9)	227 (125, 292)	80	1.80	0.179
Income lost in time caring for child	0 (0)	0	0	2 (4.8)	203 (135, 270)	96	0.01	0.933
Reduction in wealth	23 (82.1)	202 (11, 827)	202	26 (61.9)	359 (10, 1766)	485	0.06	0.806
Special home modifications	17 (60.7)	50 (3, 165)	46	23 (54.8)	232 (3, 837)	233	1.12	0.286
**Total cost of illness**	**28 (100)**	**674 (99, 2731)**	**610**	**42 (100)**	**1,132 (10, 3921)**	**1,014**	**3.54**	**0.059**

### Cost by level of household income

We used the expenditure approach to estimate the annual income of the household [38]. We then used automatic Stata weights to group the households in the study according to the annual income. As shown in Table 5, the higher the level of expenditure/income of the household, the lower the mean annual cost to the household, although differences were not significant. Care for children with developmental disabilities on average costs households with the lowest level of income more than it does the households with medium and highest income levels.

**Table 5:** Annual cost (USD $) of caring for a child with developmental disability by level of household expenditure (N = 70).

	Low Income			Medium Income			High Income				
***Variable name***	**n**	**Mean (range)**	**SD**	**n**	**Mean (range)**	**SD**	**n**	**Mean (range)**	**SD**	**Kruskal-Wallis H**	**P-value**
Cost of seeking care	24	540 (50, 2628)	649	23	523 (11, 2097)	577	22	521 (28, 2479)	557	0.12	0.942
Income lost due to loss of employment	3	284 (270, 292)	13	0	0	0	3	159 (135, 195)	32	0.04	0.982
Income lost in time caring for child	1	270 (270, 270)	0	0	0	0	1	135 (135, 135)	0	0.25	0.883
Reduction in wealth	21	179 (14, 557)	179	14	382 (10, 1766)	561	14	236 (10, 1469)	421	5.10	0.078
Special home modifications	14	18 (3, 681)	177	10	220 (8, 781)	218	16	128 (3, 84)	210	0.55	0.758
**Total cost of illness**	**24**	**998 (187, 3191)**	**810**	**23**	**958 (11, 2920)**	**916**	**23**	**892 (10, 3921)**	**1000**	**0.82**	**0.663**

### The cost of abandonment

The overall average cost of abandonment was USD 430 (range USD 7–2,920). Although the categorization is irrelevant to this sub-study, the households in the intervention arm of the Baby Ubuntu feasibility trial reported a significantly lower cost of abandonment than the households in the control arm (USD 120 (range, USD 16–235) vs. USD 740 (range, USD 7–2,920) respectively, p = 0.040).

## Discussion

Our cost of illness study estimated the annual economic burden incurred by families as a result of caring for a young child with an early developmental disability in Uganda and is one of the first to report on cost of illness to households in the region. The annual economic burden incurred by families was high and unaffordable for all participating families. Costs were higher for households in the lowest socioeconomic group, those with a child with severe neurodevelopmental impairment and those who experienced paternal abandonment. Whilst abandonment was common and associated with a negative financial impact on remaining caregivers, enrolment to a programme of early care and support for affected children and caregivers appeared to partially mitigate this risk.

In our study the annual cost of caring for a young child with a developmental disability was estimated at 949 USD as an average across our sample. The biggest cost drivers were the indirect productivity losses including income lost due to loss of employment, and the cost of seeking medical care. The former is consistent with previous studies which reported the indirect productivity losses as the biggest cost driver [9,17–20].

Earlier reviews have confirmed that parents caring a child with CP incur a larger socio-economic burden relating to that care than those whose children are not affected [21,23]. An understanding of the size and magnitude of this economic burden, most frequently mothers, can be contextualized by looking at the costs of caring in relation to the national income [36]. Households caring for children with developmental disabilities generally spend an annual average of between USD 981 and USD 2,508 more than the national average [39]. Our findings show that the average annual expenditure was more than 100% of Uganda’s GDP per capita for all the households in our sample, and in all income groups. This suggests that caring for children with developmental disabilities was unaffordable to all participating households. Moreover, we found at least 84% of households faced catastrophic expenditure leading to coping strategies such as selling their property, reducing food consumption, and/or borrowing money. In addition, we showed higher costs for urban households: on average, an urban household spent USD 452 more annually than a rural household. This may reflect increased accessibility of specialized and non-specialized health care services in urban areas.

Our study has highlighted several important factors that exacerbate the financial impact of child disability on families including socioeconomic status, the severity of the disability and paternal abandonment. Poorer households had a higher cost than relatively wealthier households. This is consistent with research on the economic burden of other diseases and conditions [35]. Whilst catastrophic expenditure occurred for all households, equitable distribution of mitigating interventions to the most marginalised families, such as poorer, single-parent families and those with children with severe disability, remains important. Secondly, our findings showed that families caring for a child with severe disability spend around USD 358 more annually than those whose child had more mild-moderate impairment. This is likely related to the escalating costs of care for children with the severest of disabilities as well as greater loss of potential earnings due to increased care needs. Social-economic status and severity of disability are likely to be important points of consideration for policy makers, healthcare providers and programme planners in seeking more equitable distribution of care for children with developmental disability and their families.

Finally, we have also shown that paternal abandonment, common amongst recruited families, substantially impacts financial status. This is consistent with a review, published in 2012, that showed that single women caring for a child with disability faced worse employment consequences than their non-single counterparts [23]. Of note, however, in our study, financial impacts appeared less in those receiving a participatory programme of early care and support. This may have been mediated by improved knowledge and understanding around childhood disability amongst fathers and extended family members when an early intervention programme is received. Further studies are needed to fully understand if early intervention programmes may mitigate financial risk to families and inform future early child disability programmes and policy.

Our findings highlight the need for disability and/or welfare benefits for affected households at risk of catastrophic healthcare expenditure due to the financial impacts of childhood disability. We provide evidence that giving special consideration to families with a child with disability when planning for national health insurance schemes in LMICs such as Uganda is warranted. Moreover, as Uganda moves towards Universal Health Coverage, mainstreaming health insurance and determining the optimal insurance premium(s), discussions on how to achieve this target in an equitable way across the population that reduces/prevents catastrophic health spending for special/marginalized groups like the families caring for children with CP should be started. Information on the full economic and social impact of CP over the life course is very important as Uganda moves towards this goal of Universal Health Coverage and mainstreaming health insurance. This information is key in determining the optimal insurance premium(s), and in discussions on how to achieve Universal Health Coverage in an equitable way across the population, and that reduces/prevents catastrophic health spending for special/marginalized groups like the families caring for children with developmental disabilities like CP.

### Limitations of our study

We interviewed caregivers of children participating in an intervention trial, who were between 12–24 months old. The CoI associated with child developmental disability has been shown to vary according to age [9]. We would expect the annual cost of caring for an older child, and or an adult with disability, to be higher than the results our study reports [9,10].

The interpretation of some findings would benefit from a qualitative methods study examining, for example, the reasons for the difference in the cost of abandonment when early care and support has been received. Such studies may support scale up of early care and support programmes, such as Baby Ubuntu.

Our study was limited to estimating the annual costs incurred and did not model the lifetime costs related to caring. Lifetime costs would provide a more complete estimate of the economic burden to families, which is useful for planning purposes by health and social care decision makers and providers.

## Conclusion

In our study, the total annual costs to households of caring for a young child with developmental disability were high and unaffordable for all families; the largest cost drivers being income loss and the cost of seeking care. Both urban and rural households faced catastrophic expenditure, regardless of level of household income. Financial impacts were exacerbated by poor socioeconomic status, severity of neurodevelopmental impairment and paternal abandonment. However, programmes providing early care and support may reduce financial impacts and mitigate financial risks. National efforts to curb this catastrophic expenditure, provide social protection and make caring for children with developmental disabilities affordable, are urgently needed. Our study provides an important starting point to estimating the magnitude of the annual economic burden incurred by households of children with development disabilities in Uganda. Studies examining the lifetime economic impact of childhood developmental disability should be prioritized, aiming to give a full picture of economic burden, provide more accurate estimates of societal economic cost and provide vital information in determining equitable and affordable access to care for all families.

## Supporting information

S1 Checklist STROBE statement—checklist of items that should be included in reports of *cross-sectional studies*. (PDF)

S1 Text User costing questionnaire. (PDF)
