# Supplementary material for: The economic burden incurred by families caring for a young child with developmental disability in Uganda
Source: PLOS Glob Public Health. 2023 Apr 19;3(4):e0000953. doi: 10.1371/journal.pgph.0000953 (PMC10115281; doi:10.1371/journal.pgph.0000953)
Supplement: S1 Text — (PDF) [file pgph.0000953.s002.pdf]

Participant ID:

____

Date of Interview:

Site (__________________________) **Group** (__ __) ________/ __________/_____________

DD/MMM/YYYY

MRC/UVRI and LSHTM Uganda Research Unit

THE COST OF NEURODEVELOPMENTAL DELAY AND DISABILITY IN UGANDA

USER COSTING QUESTIONNAIRE

PART 1: GENERAL INFORMATION

1. Date of Interview _____/ _____/ __________
   Ennaku z'omwezi *DD / MM / YYYY*

2. Child unique I.D number ___________________________
   Nnamba ya nakyewa ey'enjawulo

3. Date of birth _____/ _____/ __________
   Ennaku z'omwezi lweyazaalibwa ***(write 99 for day, 99 for month or 9999 for year if unknown)***

4. Sex of the Child (***Omwana muwala, oba mulenzi***) ……………………………………………………………………

5. District (***disitulikiti***) ……………………………………………………………………

6. What condition does the child suffer from?

   a. Neurodev't Delay (***Okukula empoola***) _____
   b. Neurodev't Disability (***Obukosefu ku bwongo***) _____
   c. Others (***Obulwade obulala***) _____

8. Completed by:

____

7. **Was written informed consent provided?** _____ *(Tick if Yes)*

PART 2: COST OF SEEKING CARE

SHORT ORAL CHILD HISTORY:

Describe the condition(s) for which you sought care in the last **three months**, and where you went. ***Nsaba onnyinyonnyole embeera y'obulamu gyewalimu eyakuviirako okugenda okufuna obujjanjabi mu bbanga eryemyezi 3 egiyise, era ombuulire wa gyewagenda.***
(This section is meant to help guide the following sections, and provide some further information for interviewer, however this section is not intended to be digitized)

9. When did you receive the diagnosis for your child?
Ddi lwebaakebera omwana nebazuula ngalina obulwadde?

8.1 Neurodev't Delay ***(Okukula empoola***)................................._____ / _____ / _________
8.2 Neurodev't Disability ***(Obukosefu ku bwongo***)..................._____ / _____ / _________
8.3 Other ***(Obulala***).._____ /______ /_________

10. Have you sought care or treatment in the last **3 months?**
Mwagendako okufuna obujjanjabi mu bbanga eryemyezi 3 egiyise?
.... _______

If no, skip to part 3

0= No ***(nedda)***
1= Yes ***(yee)***

11. If yes, how many times have you been to the hospital in the last **3 months?**
Oba yee, mirundi emeka jemugenze mu ddwaliro oba awalala awajjanabirwa mu bbanga eryemyezi 3 egiyise?

Provider	Number of Visits (Total)	Number of Visits (Delay + Disability)	Number of Visits (Other)
11.1 Mulago/Kiwoko Hospital ***Ewajjanjabirwa abasawo b'edwaliro ly'e Mulago oba Kiwoko***	________	________	________
11.2 Private Facility **Mu ddwaliro ly'obwananyini (pulayiveti)**	________	________	_________
11.3 Pharmacy ***Mu maduuka amanene agatunda eddagala***	_________	_________	_________
11.4 Traditional Healer ***Mu basawo abekinnansi***	_________	__________	___________
11.5 Herbalist ***Mu basawo abagaba eddagala ery'emiti***	_________	__________	__________
11.6 Other Government Facility or Hospital as an **Outpatient** ***Mu ddwaliro lya gavumenti Eddala ng'ogendayo okuva eka***	__________	__________	___________
11.7 Other	__________	__________	___________

Participant ID: _____________________________ **Date of Interview**: ___________________________ DD/MMM/YYYY

12. Hospital Visited

12. 1 Mulago/ Kiwoko Hospital (a) ____
12.2 Private Facility (b) ____
12.3 Pharmacy (c) ____
12.4 Traditional Healer (d) ____
12.5 Herbalist (e) ____
12.6 Other Government Facility (f) ____
12.7 Other (g) ____

13. How many times have you visited **the facility** in the last 3 months?
Mirundi emeka jemugenze awajjanjabirwa eyo mu myezi 3 egiyise?
.... ***Emirundi*** _______ times
Check with PART 2, Question 11.1 for consistency

13.1 Were the visits to this facility outpatient or inpatient?

a. Outpatient _____ (Fill qns 15 – 25)
b. Inpatient _____ (Fill qns 27 – 43)

14. What is the main mode of transport used on your most recent visit to this facility?
Ntambula ki gy'osembyeyo okukozesa ng'ojja ku ddwaliro lino okujjanjabwa mukiseera kyokyasembyeyo okujja? .... _____

1 – Walk , 2 – Bicycle, 3 – Motorbike, 4 – Bus/Taxi, 5 – Car, 6 – Bodaboda, 7 – Other

15. What is the distance from your home / workplace to the facility?
Waliwo buwanvu ki okuva ewammwe/gyokolera okutuuka ku ddwaliro?
...._____kms
(Write 99 if don't know)

16. What was the total cost of transportation (round trip) for your **most recent** visit to this **facility?** (Include costs of referral)
Wafulumya sente mmeka ku lugendo lw'okuddwaliro amagenda n'amadda ku mulundi Gwe mukyasembyeyo okujja ku ddwaliro lino okufuna obujjanjabi ng'otwaliddemu n'ezeeyo gyebali bakusindise?
……………………………………………………………………………………………UGS ______________
(Write 99999999 if don't know)

17. Indicate the **time** (in total) spend on your **most recent visit** to the hospital:
Nsaba olage obudde bwonna awamu bwe bwamala nga muzze ku ddwaliro okujjanjabwa omulundi ogukyasembyeyo.

17.1. How long did it take you to travel to and from the facility from your home / workplace?
Kyakutwalira budde ki mukugenda n'amaada wakati w'okuuva awaka oba kumulimu ne ku ddwaliro?..._______minutes

17.2. How long did you wait in the queue before being seen?
Kyabatwalira budde ki ku ddwaliro nga mulinda okubakola ko oba okulaba omujanjabi?
………………………………………………………………………………………………………. _______minutes

17.3. How long was your consultation (including referral if you went directly to another facility)?
Kyabatwalira budde ki okumaliriza okwebuuza ku musawo, ng'otwaliddemu neby'okugenda gyebakusindise bwekiba nti wagendayo butereevu? ………………………._______minutes

18. How many **adults** accompanied you to the facility on your most recent visit?
Abantu bameka abakulu abaakuwerekerako ku ddwaliro ku mulundi gwokyasembyeyo okujja okufuna obujjanjabi? ...._____ people
If 0, skip to question 20

19. For the first three **adults** accompanying you on this visit, what is their primary employment?
Mbulira emirimu gyabantu basatu kwabo abakulu abakuwelekera mu ddwaliro ku mulundi guno.

19.1. Adult 1 (***Omuntu omukulu asoka)***.. _____

19.2. Adult 2 (***Omuntu omukulu owokubiri)***.. _____

19.3. Adult 3 (***Omuntu omukulu owokusatu)*** .... _____

Codes for Employment

1= Employed by government
Akolera gavumenti

2= Employed by private sector
Akolera Bannekolera gyange

3= Employed by NGO
Akolera kitongole kya bwannakyewa

4= Employed by a private household
Akolera amaka ag'obwannanyini

5= Self-employed (merchant), business with employees
Yeekozesa (musuubuzi alina omulimu okuli n'abakozi)

6= Self-employed (merchant), business no employees
Yeekozesa yekka (musuubuzi), omulimu teguliiko bakozi

7= Working on household farm or with household livestock
Akola ku nnimiro y'awaka /ddundiro ly'awaka omuli ebisolo by'awaka.

8= Unemployed (able to work / looking for work)
Talina mulimu (asobola okukola/anoonya mulimu)

9= Unemployed (unable to work / not looking for work)
Talina mulimu (tasobola kukola/tanoonya mulimu

10 = Student
Muyizi

11 = Apprentice
Akola mirimo gyakupatana

12 = Homemaker
Akola mirimo gy'awaka

13 = Retired
Yawummula ebyokukola'

14 = I don't know
Simanyi

15 = Other(specify)________________
Ekirala (nnyonnyo

20. What was the total cost paid at the facility for this visit?
Awamu, wasasula sente mmeka ku ddwaliro kumulundi gunno?
....UGS_______________

21. How much did you pay for **lab** costs during this visit? (eg. was any blood or urine sample taken?
Wasasula sente mmeka olw'ekkeberero ly'ebyobulamu eryomuddwaliro ku mulundi ogwo (laabu)? (waliwo omusulo oba omusaayi ebyakujjibwako)?
……………………………………………………………………………………………………UGS ________________

22. How much did you pay for **diagnostic / lab** costs which were ordered during this visit, but obtained outside of the facility?
Wasasula sente mmeka *okukeberebwa****/mu*** *laabu* ***ezaakusabibwa kw'olwo lwewajja naye ebyo ate n'obikolera mu kifo kirala awatali ku ddwaliro eryo***?
……………………………………………………………………………………………………UGS ________________

23. How much did you spend on **medicines** during this visit?
Sente mmeka zewafulumya ku *ddagala* ***ku mulundi ogwo lwemwajja ku ddwaliro?***
……………………………………………………………………………………………………UGS ________________

24. How much did you spend on medicines which were prescribed during this visit, but purchased elsewhere?
Sente mmeka zewafulumya ku ddagala lyebaakuwandiikira kw'olwo naye n'oligula walala?
……………………………………………………………………………………………………UGS ________________

25. How much did you spend on **consultation fees** during this visit?
Wafulumya sente mmeka kukwebuuza ku basawo ku mulundi ogwo gwemuzze ku ddwaliro?
……………………………………………………………………………………………………UGS ________________

25. How much did you spend on **any other fees or gifts** during this visit?
Wafulumya sente mmeka ku *bintu ebirala oba ebirabo* ***kw'olwo lwemwajja ku ddwaliro?***
……………………………………………………………………………………………………UGS ________________

InPatient

27. How many times has the child been **hospitalized** as an **Inpatient** due to complications or disease symptoms in the last **three months**?
Mirundi emeka gyebawadde owana ekitanda mu ddwaliro mu bbanga eryemyezi 3 egiyise?
....______times

28. How many nights did the child stay as an Inpatient for each **admission** to the facility as an Inpatient?
Omwana yammala ebiro bimeka mu ddwaliro nga bamuwadde ekitanda ku buli mulundi lwebaakimuwanga ku nsonga y'obulwadde?

28.1. First admission

omulundi ogwasooka okumuwa ekitanda...ebiro _____ nights

28.2. Second admission

omulundi ogwokubiri okumuwa ekitanda..............................................ebiro_____ nights

28.3. Third admission

omulundi ogwokusatu okumuwa ekitanda ....ebiro _____ nights

28.4. Fourth admission

Omulundi ogwokuna okumuwa ekitanda ....ebiro _____ nights

29. What is the main mode of transport used on your **most recent** admission to the facility for treatment?
    Wasinga kweyambisa ntambula ki kumulundi gwokyasembyeyo okuweebwa ekitanda mu ddwaliro ngolwadde?

    ...._____

    Codes for transport modes

    1 = Walk (***kutambula***)
    2 = Bicycle (***ggaali***)
    3 = Motorbike (***ka pikipiki***)
    4 = Bus/Taxi (***baasi***/***takisi***)
    5 = Car (***akamotoka akaabuyonjo***)
    6 = Boda-boda (***boda boda***)
    7 = Other (specify***): ekirala (nnyonnyola)***
    ____

30. What is the distance from your home / workplace to the facility?
    Olugendo oluliwo okuva ewammwe/gyokolera okutuuka ku ddwaliro luliko buwanvu ki?

    ...._____kms

31. What was the total cost of transportation (round trip) for your **most recent** visit to this **facility** for treatment (include costs of referral if mentioned above)**?**
    Wafulumya sente mmeka zonna awamu kuntambula amagenda namadda ku mulundi gwokyasembye okugenda ku ddwaliro lino ku nsonga yobulwadde?

    ....UGS _______________
    (Write 99999999 if don't know)

32. Indicate the time (in total) spend **on your most r**ecent visit for treatment:
    Nsaba olage obudde bwonna awamu bwewamala ku mulundi gwokyasembye okugenda ku ddwaliro ku nsonga yobulwadde

    32.1. For admission, how long did it take you to travel to the facility from your home / workplace?
    Omwana lwewaweebwa ekitanda, kyakutwalila banga ki okutuuka ku ddwaliro okuva awaka oba wokolela?.._______minutes

    32.2. For discharge, how long did it take you to travel to the facility from your home?
    Omwana lwebamusibula kyakutwalira banga ki okuva ku ddwaliro okutuka awaka?
    ………..._______minutes

33. In total, (add all days from friends/relatives), how many days did your friends and/or relatives visit or accompany you while you the child an Inpatient at the facility?
    Awamu, (nga ogase enaku zzona abemikwano n'abenganda zo), zali enaku mmeka abemikwano n'abenganda zo zebakukyalila oba okujanjaba nga omwana ali kukitanda mu ddwaliro?

    ………..._______ days
    If 0, skip to question 35

34. For the first four **adults** visiting or accompanying you during this Inpatient stay, what is their primary employment?
Nsaba ombulile emirimu jja'abantu abaana kwabo abakulu abakukyalila nga oba okujanjaba nga oli ku kitanda

	**Adult 1**	**Adult 2**	**Adult 3**	**Adult 4**
Days visited ***Ennaku mmeka zeyakyala***	34.1. ________	34.2. ________	34.3. ________	34.4. ________
Primary job ***Omulimu gwe omukulu***	34.5. ________	34.6. ________	34.7. ________	34.8. ________
If other, specify: ***Omulala, nyonyola:***	34.9.	34.10.	34.11.	34.12.

Codes for Employment

1= Employed by government
Akolera gavumenti
2= Employed by private sector
Akolera Bannekolera gyange
3= Employed by NGO
Akolera kitongole kya bwannakyewa
4= Employed by a private household
Akolera amaka ag'obwannanyini
5= Self-employed (merchant), business with employees
Yeekozesa (musuubuzi alina omulimu okuli n'abakozi)
6= Self-employed (merchant), business no employees
Yeekozesa yekka (musuubuzi), omulimu teguliiko bakozi
7= Working on household farm or with household livestock
Akola ku nnimiro y'awaka /ddundiro ly'awaka omuli ebisolo by'awaka.
8= Unemployed (able to work / looking for work)
Talina mulimu (asobola okukola/anoonya mulimu)
9= Unemployed (unable to work / not looking for work)
Talina mulimu (tasobola kukola/tanoonya mulimu
10 = Student
Muyizi
11 = Apprentice
Akola mirimo gyakupatana
12 = Homemaker
Akola mirimo gy'awaka
13 = Retired
Yawummula ebyokukola'
14 = I don't know ***(simanyi)***
15 = Other(specify)________________
Ekirala (nnyonnyo)

35. What was the **total cost** paid at the facility for this visit?
Awaamu, wasasula sente mmeka ku ddwaliro omulundi guno?
……………………………………………………………………………………………………UGS _______________

36. How much did you pay for hospital **accommodation**, **admission** and **consultation fees**?
Wasasula sente mmeka mu ddwaliro ezokuwa owana ekitanda, okukubezaayo nezokwebuuza ku musawo? ……………………………………………………………………………UGS _______________

37. How much did you pay for **food** (either within the facility, or brought in)?
    Wasasula ssente mmeka ez'emmere wadde nga ya ddwaliro oba nga ogiguze okuva wabweru?
    ……………………………………………………………………………………………UGS _______________

38. How much did you pay for **lab** costs during this visit? (eg. was any blood or urine sample taken?)
    W***asasula sente mmeka ezekkeberero lyebyobulamu-laabu, ku mulundi ogwo? Okugeza, baakuggyako omusaayi oba omusulo***?
    ……………………………………………………………………………………………UGS _______________

39. How much did you pay for **diagnostic / lab** costs which were ordered during this visit, but obtained outside of the facility?
    Wasasula sente mmeka ezokwekebeza /eza laabu ezaakusabibwa ku mulundi ogwo, naye nobifunira walala awatali awo ku ddwaliro?
    ……………………………………………………………………………………………UGS _______________

40. How much did you spend on **medicines** during this visit?
    Wafulumya sente mmeka ku ddagala ku mulundi ogwo?
    ……………………………………………………………………………………………UGS _______________

41. How much did you spend on **medicines** which were prescribed during this visit, but purchased elsewhere?
    Wasasula sente mmeka ku ddagala lye baakuwandiikira kwolwo naye ate noligula walala?
    ……………………………………………………………………………………………UGS _______________

42. How much did you spend on **consultation fees** during this visit?
    Wafulumya sente mmeka kukwebuuza ku musawo kw'olwo?
    ……………………………………………………………………………………………UGS _______________

43. How much did you spend on **any other fees or gifts** during this visit?
    Wafulumya sente mmeka ku bintu ebirala oba ku birabo
    ……………………………………………………………………………………………UGS _______________

PART 3: SOCIO-ECONOMIC STATUS

DESCRIPTION OF INCOME ACTIVITIES

Please tell us how you make your living. This section is meant to help guide the following sections, and provide some further information for interviewer, however this section is not intended to be digitized.

Tusaba otubuulire engeri gy'oyimirizaawo obulamu bwo. Kino ekitundu kigenda kuyambako mukulambika ebitundu ebiddako era nokuwa abuuza ebibuuzo amawulire agasingawo.

44. In the past 3 months, which of the following describes your main work status?

Kiki ku bino wammanga ekinnyonnyola omulimu gw'osinze okukola mu bbanga ery'emyezi esatu egiyise?

44.1. Primary job ***(omulimu ogusinga obukulu)*** ………………………………………………… ________

44.2. Secondary job ***(omulimu omulala)*** ……………………………………………………………. ________

Codes for Employment

1= Employed by government
Akolera gavumenti

2= Employed by private sector
Akolera Bannekolera gyange

3= Employed by NGO
Akolera kitongole kya bwannakyewa

4= Employed by a private household
Akolera amaka ag'obwannanyini

5= Self-employed (merchant), business with employees
Yeekozesa (musuubuzi alina omulimu okuli n'abakozi)

6= Self-employed (merchant), business no employees
Yeekozesa yekka (musuubuzi), omulimu teguliiko bakozi

7= Working on household farm or with household livestock
Akola ku nnimiro y'awaka /ddundiro ly'awaka omuli ebisolo by'awaka.

8= Unemployed (able to work / looking for work)
Talina mulimu (asobola okukola/anoonya mulimu)

9= Unemployed (unable to work / not looking for work)
Talina mulimu (tasobola kukola/tanoonya mulimu

10 = Student
Muyizi

11 = Apprentice
Akola mirimo gyakupatana

12 = Homemaker
Akola mirimo gy'awaka

13 = Retired
Yawummula ebyokukola'

14 = I don't know ***(simanyi)***

15 = Other(specify)________________
Ekirala (nnyonnyo)

45. In the past three months, how many days on average per month did you work at each job?

Bw'oba ogerageranyizza, nnaku mmeka zewakola ku buli mulimu buli mwezi mu myezi esatu egiyise?

45.1. Primary job ***(omulimu ogusinga obukulu)*** ………………………………………… ________ days

45.2. Secondary job ***(omulimu omulala)*** …………………………………………………… ________ days

46. What was your last monthly wage (or in-kind equivalent) for each job?

Omusaala gwo ogw'omwezi oguwedde gwali gwenkana wa ku buli mulimu? (gerageranya sente ezigya mw'ekyo kyewafuna bwekiba kyali kintu kikalu.)

	a. Cash<br>***Sente mu buliwo***	**b**. Estimated value of in-kind payments<br>***Embalirira eteeberezebwa mu bintu ebikalu ebikozeseddwa mukusasulwa***
132.1 Primary Job<br>***Omulimu omukulu***	UGS ________________	UGS _________________
132.2 Secondary Job<br>***Omulimu omulala***	UGS ________________	UGS _________________

47. Please list all of the other usual members of your household. For each member, if they were employed in the last three months please list their employment.

	**a. Relationship**	**b. Adult / Child**	**c. Primary Job**
47.1. Household Member #1<br>***Mmemba womumaka gano asooka***	___________________	________	________
47.2. Household Member #2<br>***Mmemba womumaka gano owokubiri***	___________________	________	________
47.3. Household Member #3<br>***Mmemba womumaka gano owokusatu***	___________________	________	________
47.4. Household Member #4<br>***Mmemba womumaka gano owokuna***	___________________	________	________
47.5. Household Member #5<br>***Mmemba womumaka gano owokutaano***	___________________	________	________
47.6. Household Member #6<br>***Mmemba womumaka gano owomukaga***	___________________	________	________
47.7. Household Member #7<br>***Mmemba womumaka gano owomusanvu***	___________________	________	________
47.8. Household Member #8<br>***Mmemba womumaka gano owomunana***	___________________	________	________
47.9. Household Member #9<br>***Mmemba womumaka gano owomwenda***	___________________	________	________
47.10. Household Member #10<br>***Mmemba womumaka gano owekumi***	___________________	________	________

Codes for Age

1= Adult ***(Omuntu mukulu)***
2= Child ***(Mwana muto)***

Codes for Employment

1= Employed by government
Akolera gavumenti
2= Employed by private sector
Akolera Bannekolera gyange
3= Employed by NGO
Akolera kitongole kya bwannakyewa
4= Employed by a private household
Akolera amaka ag'obwannanyini
5= Self-employed (merchant), business with employees
Yeekozesa (musuubuzi alina omulimu okuli n'abakozi)
6= Self-employed (merchant), business no employees
Yeekozesa yekka (musuubuzi), omulimu teguliiko bakozi
7= Working on household farm or with household livestock
Akola ku nnimiro y'awaka /ddundiro ly'awaka omuli ebisolo by'awaka.
8= Unemployed (able to work / looking for work)
Talina mulimu (asobola okukola/anoonya mulimu)
9= Unemployed (unable to work / not looking for work)
Talina mulimu (tasobola kukola/tanoonya mulimu
10 = Student
Muyizi
11 = Apprentice
Akola mirimo gyakupatana
12 = Homemaker
Akola mirimo gy'awaka
13 = Retired
Yawummula ebyokukola'
14 = I don't know ***(simanyi)***
15 = Other(specify)________________
Ekirala (nnyonnyo)

48. Do you get income or support from any of the following sources:
Offuna ensimbi oba obuyambi mu bintu bino wamanga?

	a. Type of income / support	b. Estimated monthly value
48.1 Income source 1 ***Obuyambi obusooka***	________	UGS __________________
48.1 Income source 2 ***Obuyambi obwokubiri***	________	UGS __________________
48.2 Income source 3 ***Obuyambi obwokusatu***	________	UGS __________________

Write 99999 if don't know

Codes for column A:

1= Remittances
Sente eziweerezebwa awantu
2= Charity / church
Mu masinzizo oba abazirakisa
3= Retirement pension
Akasiimo akafunibwa ng'owumudde emirimo
4= NSSF
Sente zeweeterekera ezikuweebwa **ng'okuze**
5= Welfare grants
Obuyambi obuweebwa abali mubwetaavu
6= Bursary / study loan
Okuyambibwako mu kusasula ebisale by'essomero
7= Other (specify)
Ekirala (nnyonnyola)

49. Do you have any other source of income that hasn't been mentioned? What is the average monthly value?
Waliwo engeri endala ey'ennyingiza etayogeddwako? Okutwalira awamu zziri mmeka buli mwezi?

	**a.** Source of income ***Ekkubo ly'ennyingiza***	**b.** Estimated cash value ***Ssente ezibalirirwamu***	**c.** Estimated value of in-kind payments ***Sente ezibalirirwa* mu *bintu ebikalu ebifunibwa mukusasulwa***
49.1	_____________________	UGS _________________	UGS _________________
49.2	_____________________	UGS _________________	UGS _________________
49.3	_____________________	UGS _________________	UGS _________________
49.4	_____________________	UGS _________________	UGS _________________

Write 99999 if don't know

50. What are the total recurrent expenses of your household?

	Items of expenditure and Savings	a. Cash amount or in-kind equivalent	b. Time Period
50.1.	Food<br>***Emmere***	UGS _______________________	_________
50.2.	Transportation<br>***Entambula***	UGS _______________________	_________
50.3.	Charcoal or firewood<br>***Amanda oba enku***	UGS _______________________	_________
50.4.	Communication (phone)<br>***Essimu***	UGS _______________________	_________
50.5.	Toiletries<br>***ebikozesebwa mu kinabiro oba kabuyonjo***	UGS _______________________	_________
50.6.	Utilities (electricity)<br>***Amasanyalaze***	UGS _______________________	_________
50.7.	Utilities (gas)<br>***Gaasi***	UGS _______________________	_________
50.8.	Utilities (water)<br>***Amazzi***	UGS _______________________	_________
50.9.	House Rent<br>***Ebisale by'ennyumba***	UGS _______________________	_________
50.11.	Leisure / Recreation<br>***Ebyokwesanyusa***	UGS _______________________	_________

Write 999999 if don't know

Codes for Column B:

1 = Day (***olunaku)***
2 = Week ***(sabbiiti)***
3 = Month ***(omwezi)***
4 = Year ***(omwaka)***
5 = Other (specify)
Ekirala nnyonyola

Completed by ___________________________

51. In addition to the expenses specified above, what is the total monthly in-kind value you gain (ie. eggs, milk, meat) from any household livestock or home-grown produce?
Gerageranya sente ezigya mu bintu ebikalu okuva mu byolima oba byolunda awaka (gezesa nga amagi, amaata oba enyama)

	**a. Describe**	**b. Estimated monthly value**
51.1. Livestock<br>***Ebyobulunzi***	____________________	UGS ______________________
51.2. Home-grown produce<br>***Ebyokulima***	____________________	UGS ______________________
51.3. Charcoal / firewood<br>***Amanda/enku***	____________________	UGS ______________________
51.4. Other (specify)<br>***Ekirala(nnyonyola)***	____________________	UGS ______________________

Write 99999 if don't know

52. Do you have any form of health insurance or other medical aid?
Olina Yinsuwa y'obulamu oba engeri endala yonna ekuyamba kuby'obujjanjabi?............. _________
0= No
1= Yes

53. What is the monthly / annual premium that **you pay** for the health insurance (do not include additional premiums payed by employer/govt etc)?
Sente mmeka z'osasula buli mwezi/omwaka ku yinsuwa y'obulamu (nga totwliddeemu ezikwongerwako oyo okukozesa/gavumenti oba ebirala)
…………………………………………………………………………………………………..UGS__________________________
(write 99999999 if don't know)

54. Does this insurance cover all or only part of your health costs?
Yinsuwa eno esasulira eby'obulamu bwo byonna oba esasulirako bimu?

……..._________

1= All costs (***esasulira byonna***)
2= Partial costs (***esasulirako bimu***)

55. If partial costs, what percentage?
Bwebiba byakitundu, esasulirako bitundu bimeka ku kikumi?....................................._____________%

PART 4: COST OF ILLNESS

56. After your child was diagnosed, did you have a change in employment (ie. Lost job or change in job) as a result of symptoms associated with or treatment?
Mu myezi essatu egiyise wafunamu enkyukakyuka ku mulimu nga kiva ku bubonero bwobulwadde mu mwana?
....________

If no, go to question 60

0= No ***(Nedda)***
1= Yes ***(Yee)***

57. Do you remember the date of this change in employment?
Ojjukira ddi enkyukakyuka eno ku mulimu lweyaliwo?
.... ________ /________ / ______________
DD/MM/YYYY
If don't know, put 99/999/9999)

58. If yes, did this change in employment result in a loss or gain in income?
Oba yee, enkyukakyuka eno yavaamu okukufiiriza ora wagiganyulwamu ku ludda lwe nnyingizas?
....________

1= Loss in income ***(wafiirizibwa munnyingiza)***
2= Gain in income ***(waganyulwamu mu nnyingiza)***

59. What is the average monthly value of this loss/gain?
Bwogerageranyiza kyewafiirizibwa/kyewaganyulwa buli mwezi kiba kyenkanawa?
....UGS ___________________________

60. In the last three months, how many days on average per month did you need to take off from work in total due to feeling unwell or using health services as a result of symptoms associated sickeness?
Mu bbanga lya myezi 3 egiyise, nnaku mmeka buli mwezi mu kugerageranya zewava ku mulimu nga teweewulira bulungi oba nga wetaaga okufuna obujjanjabi olw'bulwadde?
....___________ days
If zero, go to question 64

61. The last time you needed to take time off from work, which job did you need to take off from?
Omulundi gwokyasembye okwagala okuva ku mulimu, gwali mulimu ki gwewali ovaako?
....________

1 = My primary job ***Ku mulimu gwange omukulu***
2 = My secondary job ***Ku mulimu gwange owokubiri***
3 = A different job not already mentioned ***Ku mulimu omulala ogutannayogerwako wano***

62. If a different job not already mentioned, what is the main occupation category for this job?
Oba mulimu mulala ogutannayogerwako wano, gugwa mu ttuluba ki?

...._________

Codes for Employment

1= Employed by government
Akolera gavumenti
2= Employed by private sector
Akolera Bannekolera gyange
3= Employed by NGO
Akolera kitongole kya bwannakyewa
4= Employed by a private household
Akolera amaka ag'obwannanyini
5= Self-employed (merchant), business with employees
Yeekozesa (musuubuzi alina omulimu okuli n'abakozi)
6= Self-employed (merchant), business no employees
Yeekozesa yekka (musuubuzi), omulimu teguliiko bakozi
7= Working on household farm or with household livestock
Akola ku nnimiro y'awaka /ddundiro ly'awaka omuli ebisolo by'awaka.
8= Unemployed (able to work / looking for work)
Talina mulimu (asobola okukola/anoonya mulimu)
9= Unemployed (unable to work / not looking for work)
Talina mulimu (tasobola kukola/tanoonya mulimu
10 = Student
Muyizi
11 = Apprentice
Akola mirimo gyakupatana
12 = Homemaker
Akola mirimo gy'awaka
13 = Retired
Yawummula ebyokukola'
14 = I don't know ***(simanyi)***
15 = Other(specify)________________
Ekirala (nnyonnyo)

63. How much income (cash, or in-kind equivalent) would you have expected to make on the days you were unable to work?
Ssente mmeka mu buliwo oba mu bintu ebikalu zewali osuubira okufuna mu nnaku ezo zootaasobola kukola mirimo? ....UGS ___________________________
(write 99999999 if don't know)

64. After the diagnosis, was there anyone who covered your normal work duties as a result of you taking care of your child?
Nga bamaze okuzuula obulwadde mumwanawo, waliwo omuntu eyakukolerako emirimo gye waandikoze, gwe ng'ojjanjaba omwanawo?

....__________

If no, go to question 67

0= No (***Nedda***)
1= Yes (***yee***)

65. Approximately how many days per month did this person work in your stead after the diagnosis?
Nnaku nga mmeka buli mwezi mukugerageranya omuntu ono zeyakola mu kifo kyo oluvannyuma lwokuzuula obulwadde obwo mumwana?

....____________ days

66. Approximately how much did you pay this person per day?
    Mukugeragerenya, omuntu ono wamusasulanga sente mmeka buli lunaku?
    ……………………………………………………………………………………UGS ___________________________
    (write 99999 if don't know, 00000 if not paid)

67. After your child's diagnosis, did you stop performing any of your normal household duties due to taking care of your child (includes any cooking, cleaning, collecting firewood / water, fishing/hunting, farming, etc.)?
    Nga bamaze okukubuulira obulwadde bw'omwanawo, walekayo emirimo egimu egy'awaka olw'obuteewulira bulungi oba olwokuba walina okumujjanjaba? Mu bino mulimu, okufumba, okuyonja, okusennya enku/ amazzi, okuvuba, okuyigga, oba okukola mu nnimiro n'ebirala ………_________
    If no, go to question 71
    0 = No (***nedda***), 1 = Yes (***yee***)

68. Is there anyone else in your household who can take over your household duties when you are unable to do them?
    Awaka wammwe waliwo omuntu omulala asobola okukola emirimu egya'waka singa ggwe oba tosobola kubikola? ……………………………………………………………………………………………………_________
    0 = No (***nedda***)
    1 = Yes (***yee***)

69. If so, how many days per month had to be covered by this person because you were unable to do your household tasks?
    Bwekiba kityo, nnaku mmeka buli mwezi omuntu oyo zeyakuyambako olw'ensonga nti gwe wali tosobola kukola mirimo gyo egyawaka?……………………………………………………………_________days

70. What is the main occupation of this person?
    Omuntu oyo akola mulimu ki omukulu? ……………………………………………………………………_________

Codes for Employment

1= Employed by government
Akolera gavumenti
2= Employed by private sector
Akolera Bannekolera gyange
3= Employed by NGO
Akolera kitongole kya bwannakyewa
4= Employed by a private household
Akolera amaka ag'obwannanyini
5= Self-employed (merchant), business with employees
Yeekozesa (musuubuzi alina omulimu okuli n'abakozi)
6= Self-employed (merchant), business no employees
Yeekozesa yekka (musuubuzi), omulimu teguliiko bakozi
7= Working on household farm or with household livestock
Akola ku nnimiro y'awaka /ddundiro ly'awaka omuli ebisolo by'awaka.

8= Unemployed (able to work / looking for work)
Talina mulimu (asobola okukola/anoonya mulimu)
9= Unemployed (unable to work / not looking for work)
Talina mulimu (tasobola kukola/tanoonya mulimu
10 = Student
Muyizi
11 = Apprentice
Akola mirimo gyakupatana
12 = Homemaker
Akola mirimo gy'awaka
13 = Retired
Yawummula ebyokukola'
14 = I don't know ***(simanyi)***
15 = Other(specify)________________
Ekirala (nnyonnyo)

71. After your child's diagnosis, did you have to sell anything to pay for any health services or to cover for any loss of income due to feeling unwell, or using health services?
Walina okutunda ebimu ku bibyo osobole okusasula ebyobujjanjabi oba okusasulira ebitagenze bulungi mubyenfuna olw'okujjanjaba omwanawo?

....__________

If no, go to question 73

0= No
1= Yes

72. What is the total value of the sold goods?
Gerageranya mu ombulire ensimbi ezigya mu muwendo gwebintu byewatunda

....UGS____________________________

(write 99999999 if don't know)

73. After your child's diagnosis, did you have to borrow any money to pay for any health services or to cover for any loss of income due to taking care of your child?
Walina okwewola sente okusobola okusasulira ebintu ebimu oba okuziba ebituli mubyennyingiza Olw'okujjanjaba omwanawo?

....__________

If no, go to question 76

0= No
1= Yes

74. How much did you have to borrow in total?
Sente zonna awamu zewalina okwewola zaali mmeka?

....UGS____________________________

(write 99999999 if don't know)

175. How much in total do you have to pay back?
Sente zonna awamu zolina okusasula ziri mmeka?

....UGS____________________________

(write 99999999 if don't know)

76. After your child's diagnosis, did your household need to reduce food consumption in order to pay for any health services or to cover for any loss of income due to taking care of your child?
Nga bamaze okuzuula obulwadde bw'omwanawo, abomumaka gammwe baalina okukendeeza ku mmere eriibwa olwokusobola okufissa sente okusasulira obujjanjabi oba okusasulira ebyenfuna ebyayononeka olwokujjanjaba omwanawo?

……_________

If no, go to question 78

0= No
1= Yes

77. If yes, by how much did you reduce spending on food per month?
Oba yee, mwakendeeza kyenkanawa ku mmere buli mwezi?

……………………………………………………………………………………………UGS____________________________

(write 99999999 if don't know)

78. Describe any other expenses you've incurred in the past year due to special accommodations or home refurbishment as a result of your child's condition, such as wheel chair, special home facilities, special diets, etc.
Nsaba onnyinyonnyole enfulumya endala yonna gyewakola mu mwaka oguwedde olw'ensonga y'okufuna ebikozesebwa oba okuzza obujja ebyo by'olina olw'ensonga y'embeera y'obulamu bwomwanawo, gamba nga okufuna akagaali, ebintu eby'enjawulo ebyawaka, ebyokulya ebyenjawulo, nebirala?

……………………………………………………………………………………………UGS ____________________________

(write 99999999 if don't know)

PART 5: COST OF ABANDONMENT

79. Did you have a partner before your child was diagnosed?
    Ng'omwana tanafuna bulwadde, walina omubezi gwewali obeela naye?

    ....________

    0= No ***(Nedda)***
    1= Yes ***(Yee)***

80. After the diagnosis, did your partner change the amount of support given?
    Bwemwalaba obubonero bwobulwadde mu mwana, omubeezi wo yakyusamu mu mbela y'okukuwa obuyambi?

    ....________

    0= No ***(Nedda)***
    1= Yes ***(Yee)***

81. Do you remember the date of this change in support?
    Ojjukira ddi enkyukakyuka eno mu buyambi lweyaliwo?
    .... ________ /________ / ______________
    DD/MM/YYYY
    If don't know, put 99/999/9999)

82. If yes, did this change in support result in a loss or gain in income?
    Oba yee, enkyukakyuka eno yavaamu okukufiiriza oba wagiganyulwamu ku ludda lwe nnyingizas?
    ....________

    1= Loss in income ***(wafiirizibwa munnyingiza)***
    2= Gain in income ***(waganyulwamu mu nnyingiza)***

83. What is the average monthly value of this loss/gain?
    Bwogerageranyiza kyewafiirizibwa/kyewaganyulwa buli mwezi kiba kyenkanawa?
    ....UGS ___________________________

PART 6: CONCLUSION

84. We have come to the end of this questionnaire but before I leave I wonder if you have any other comments about the costs of your health care that you would like me to record.
Tutuuse ku nkomerero y'ebibuuzo bino, naye nga ssinnagenda, simanyi oba olina ekirala kyewandyagadde okwogera ku nfulumyayo ku by'obulamu bwo nga wandyagadde nkitwale.

____ Yes ***(Yee)*** ____ No ***(Nedda)***

85. Do you have any questions for me?
Olina ekibuuzo kyonna gyendi?

____ Yes ***(Yee)*** ____ No ***(Nedda)***

Completed by ___________________________
